# Beta2-Adaptin Binds Actopaxin and Regulates Cell Spreading, Migration and Matrix Degradation

**DOI:** 10.1371/journal.pone.0046228

**Published:** 2012-10-02

**Authors:** Jeanine Pignatelli, Matthew C. Jones, David P. LaLonde, Christopher E. Turner

**Affiliations:** 1 Department of Cell and Developmental Biology, State University of New York, Upstate Medical University, Syracuse, New York, United States of America; 2 Weill Institute for Cell and Molecular Biology, Cornell University, Ithaca, New York, United States of America; Thomas Jefferson University, United States of America

## Abstract

Cell adhesion to the extracellular matrix is a key event in cell migration and invasion and endocytic trafficking of adhesion receptors and signaling proteins plays a major role in regulating these processes. Beta2-adaptin is a subunit of the AP-2 complex and is involved in clathrin-mediated endocytosis. Herein, β2-adaptin is shown to bind to the focal adhesion protein actopaxin and localize to focal adhesions during cells spreading in an actopaxin dependent manner. Furthermore, β2-adaptin is enriched in adhesions at the leading edge of migrating cells and depletion of β2-adaptin by RNAi increases cell spreading and inhibits directional cell migration via a loss of cellular polarity. Knockdown of β2-adaptin in both U2OS osteosarcoma cells and MCF10A normal breast epithelial cells promotes the formation of matrix degrading invadopodia, adhesion structures linked to invasive migration in cancer cells. These data therefore suggest that actopaxin-dependent recruitment of the AP-2 complex, via an interaction with β2-adaptin, to focal adhesions mediates cell polarity and migration and that β2-adaptin may control the balance between the formation of normal cell adhesions and invasive adhesion structures.

## Introduction

Directed cell migration is a highly coordinated and dynamic process that involves polarization of the cell in response to an external stimulus, followed by the extension of membrane protrusions in the direction of migration [1]. Subsequent adhesion to the extracellular matrix (ECM), via the integrin family of transmembrane receptors stabilizes these protrusions [1], [2]. Upon ligand binding, integrins cluster and form multiprotein assemblies, consisting of signaling, adaptor and structural proteins that mediate physical links to the actin cytoskeleton,. These structures, called focal adhesions provide the traction forces required for efficient cell motility to occur and also act as signaling hubs that integrate the multiple regulatory pathways involved in the coordination of the cell migration machinery [2], [3].

Endocytic trafficking of activated receptors serves to compartmentalize, amplify or terminate their downstream signaling pathways. The importance of endocytic trafficking in regulating growth factor signal transduction is well established [4], [5], [6] and there is a growing literature outlining the role for endocytic trafficking of receptors in the regulation of cell migration and invasion [4], [7], [8], [9]. Clathrin-mediated endocytosis has been linked to the regulation of cell polarization during migration [10], [11] and clathrin, as well as a number of clathrin-associated scaffold proteins, has been localized to focal adhesions [10], [12]. For example, dynamin-2 is recruited to focal adhesions via an interaction with FAK [13], following generation of PIP2 at adhesions by PIPKIβ [14], where it is subsequently phosphorylated in a Src-dependent manner [15]. In addition, the adaptor protein Dab2 has been shown to facilitate microtubule-dependent focal adhesion disassembly and integrin endocytosis following nocodazole washout [16], [17].

The adaptor protein-2 complex (AP-2) is a heterotetrameric complex consisting of two large (α and β 2), one medium (μ2) and one small (σ2) subunit that is involved in clathrin-mediated endocytosis of select receptors by directly linking clathrin with cargo proteins [18]. The μ2 subunit of the AP-2 complex has been shown to localize to focal adhesions and to be enriched at the leading edge of motile cells [16], [19], suggesting that AP-2 may be involved in selecting specific cargo for endocytosis at adhesions and at the leading edge of migrating cells. Therefore, proteins associated with the regulation of clathrin-mediated endocytosis may play key roles in the regulation of adhesion turnover and signaling during adhesion-dependent events such as cell migration and invasion. It remains unclear, however, how regulators of endocytosis are recruited to sites of adhesion and how manipulating endocytosis influences adhesion-dependent signaling events.

Herein we demonstrate a novel interaction between the focal adhesion protein actopaxin (α-parvin) [22] and β2-adaptin, which is required for β2-adaptin recruitment to focal adhesions. Depletion of β2-adaptin by RNAi increases cell spreading and inhibits directional cell migration via a loss of cellular polarity. Beta2-adaptin knockdown resulted in the generation of matrix-degrading adhesions termed invadopodia, typically found in cancer cells [20], [21]. These data suggest a role for β2-adaptin in the regulation of adhesion related signaling required for directed cell migration and matrix degradation.

## Materials and Methods

### Cell Culture and Reagents

U2OS cells (ATCC) were cultured in DMEM, 10% FBS, 1 mM glutamine, 50 U/ml penicillin and 50 µg/ml streptomycin, 1 mM sodium pyruvate and kanamycin. MCF10A cells (ATCC) were cultured in DMEM/F-12 (50∶50), 15 mM HEPES pH 7.5, 2 mM L-glutamine, 50 units/ml penicillin, 50 µg/ml streptomycin, 5% horse serum, 0.02 µg/ml EGF, 0.5 µg/ml hydrocortisone and 10 µg/ml insulin. Cells were maintained at 37°C and 5% CO_2_.

The following antibodies were used for immunofluorescence and Western blotting: β-adaptin, α-adaptin, γ-adaptin, clathrin heavy chain, GM130, ILK, paxillin clone 349 (BD Transduction), paxillin clone H114, Src, GFP (Santa Cruz Biotechnology), α-actinin, actopaxin (Sigma), Src pY418, FAK pY397 (Invitrogen). The Src inhibitor, PP2 (Calbiochem), was used at 2 µM.

### Transfections

For siRNA transfections, cells were plated at 1.2×10^5^ cells per well of a 6-well dish the day before transfection. Transfections were performed using Oligfectamine (Invitrogen) according to manufactures instructions. Sequences for siRNAs (Ambion) used are as follows: human β2-adaptin 5′-CAACUUAAUUGUCCAGAAA-3′ and 5′-UUUCUGGACAAUUAAGUUG-3′; control 5′-ACUCUAUCUGCACGCUGACUU-3′ and 5′-GUCAGCGUGCAGAUAGAGUUU-3′. Cells were used 72 hrs post transfection.

### GST-binding Assay

GST binding assays were carried out as previously described [22]. Briefly, U2OS cells were lysed in GST lysis buffer (20 mM Tris/HCl pH 7.6, 50 mM NaCl, 1% NP-40, 10% glycerol, 1 mM PMSF, and 10 mg/ml leupeptin. Clarified lysates were incubated end over end at 4°C for 2 hours then washed 3x. Samples were solubilized in 2x SDS-sample buffer and evaluated by Western blotting.

### Immunoprecipitation

U2OS cells were lysed in co-immunoprecipitation buffer (20 mM Tris/HCl pH 7.6, 0.5% NP-40, 100 mM NaCl, 10% glycerol, 1 mM MgCl_2_, 10 mg/ml leupeptin, 1 mM NaF, 1 mM PMSF). Clarified lysates were incubated end over end for 2 hours at 4°C with primary antibody then an additional hour with protein A/G beads (Santa Cruz). Immunoprecipitates were solubilized in sample buffer and analyzed by Western blot.

### Western Blotting

Samples were run on 10% SDS-PAGE and transferred to nitrocellulose. Primary antibodies were incubated for 2 hours followed by 1 hour incubation on secondary HRP conjugated antibodies (Jackson Laboratories) at room temperature. Western blots were visualized by chemiluminescence using ECL (GE).

### Immunofluorescence

Coverslips were fixed in 3.7% formaldehyde and then permeabilized in 1% Triton-X-100 in PBS (phosphate buffered saline). Primary antibodies were used at 1∶250 in 3% BSA in PBS for 90 minutes at 37°C. Rhodamine phalloidin (1∶1000) (Invitrogen) was used to visualize F-actin. Secondary antibodies (Jackson Immunoresearch Laboratories) were used at 1∶250 for 1 hour at 37°C. Images were acquired on a Nikon Eclipse TE2000-U inverted microscope with a Nikon Apo oil 60x/1.40NA objective using a Spot RT Slider camera and Spot Advance software. Image analysis was performed using NIH ImageJ.

### Cell Spreading

Cells were seeded at 10,000 cells per well of a 10 µg/ml collagen coated 24 well tissue-culture dish in complete media. Time-lapse live cell images were captured every 2 minutes over a 4 hour time period on a Nikon Eclipse Ti microscope using a 10X/0.30 PL FLUOR Nikon objective and equipped with a Hamamatsu Orca R2 camera (Hamamatsu City, Japan) and Nikon NS-Elements software.

### Wound Healing

For wound healing assays, cells were plated to confluence in a 35 mm dish for migration rate analysis or on a glass coverslip for Golgi polarization. The monolayer was wounded using a pipette tip. For migration rate analysis, images were acquired every 2 hours to determine the area of wound closure. For Golgi polarization, coverslips were fixed 4 hrs post wounding and stained for GM130, paxillin, actin and DAPI.

### Gelatin Matrix Degradation

Gelatin matrix degradation assays were performed as previously described [23]. Briefly, acid-washed coverslips were coated with 50 µg/ml poly-L-lysine (Sigma) in PBS, incubated in 0.5% glutaraldehyde (Sigma) in PBS and then coated with 1∶40 488-gelatin (Invitrogen,) with 0.2% w/v unlabeled gelatin solution (Sigma) in PBS at 37°C. U2OS cells were plated for 16 hours and MCF10A cells for 6 hours in serum-containing media and coverslips were processed as above.

### Clathrin Inhibition and Transferrin Internalization

Cells were treated with 50 µm monodansylcadaverine (MDC) (Sigma) or vehicle for 1 h at 37°C. TRITC-transferrin 25 µg/ml (Invitrogen) was added to the media and cells were placed at 37°C for 20 min to allow for internalization. Cells were placed at 4°C and the surface bound transferrin was stripped using an acid wash (0.2 M NaCl, 0.2 M acetic acid in PBS). Cells were than fixed and mounted for visualization of internalized TRITC-transferrin.

For gelatin degradation, cells were plated on 488-gelatin coverslip as described above in the presence of vehicle or 50 µm MDC.

### Pearson’s Correlation

Pearson’s correlation coefficient was performed using the Image J software. Pearson’s correlation coefficients were calculated from the TRITC and Cy5 channels comparing paxillin and β-adaptin staining. The Pearson’s correlation reflects the linear relationship between the localized intensities of the fluorophore labeled proteins.

### Statistical Analysis

Values were calculated from at least 3 independent experiments and were compared by student t-test and P<0.05 was considered statistically significant. Error bars represent the standard error of the mean (SEM).

## Results

### β2-Adaptin Localizes to Focal Adhesions through an Interaction with Actopaxin

**Figure 1:**
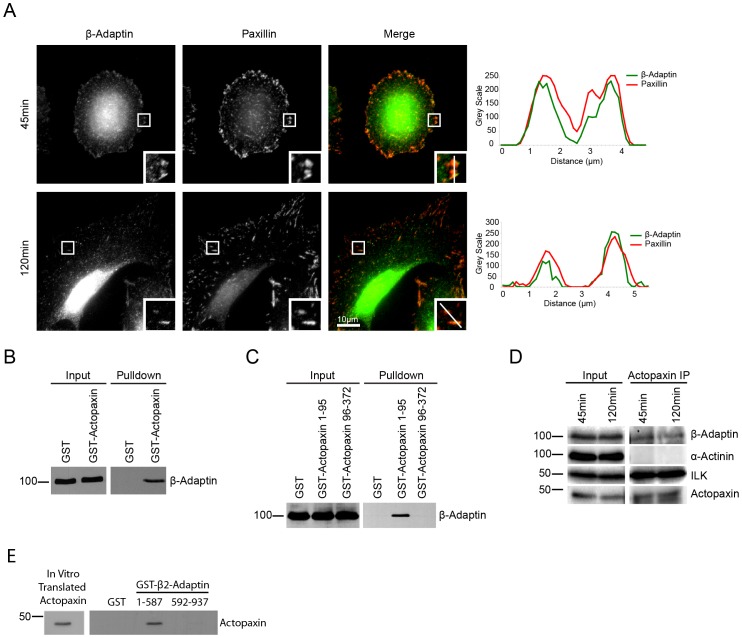
Beta 2-adaptin localizes to focal adhesions and interacts with actopaxin. **A.** U2OS cells spread on collagen for 45 and 120 min demonstrate endogenous β-adaptin localizes to paxillin containing adhesions. Line profiles demonstrate the co-localization of β-adaptin and paxillin through the center of two adhesions. Scale bar = 10 µm. **B.** GST-pulldown from U2OS cell lysates shows β-adaptin interacts with full-length actopaxin **C.** GST-pulldown of U2OS cell lysates shows β2-adaptin interacts with the N-terminal 1–95 fragment of actopaxin, but not the 96–372 fragment. **D.** Endogenous β-adaptin is co-immunoprecipitated with actopaxin when U2OS cells are spread on collagen for 45 and 120 minutes. **D.** Actopaxin directly interacts with β2-adaptin as shown by an *in vitro* transcription/translation pulldown.

Clathrin-coated pits have been shown to be enriched around focal adhesions [24] and a number of proteins associated with clathrin-mediated endocytosis can localize to focal adhesions [13], [16], [17]. Using an antibody that recognizes both the β1- and β2-adaptin components of the AP-1 and AP-2 complexes, we found that endogenous β-adaptin, which binds directly to clathrin, localizes to paxillin positive focal adhesion structures during U2OS osteosarcoma cell spreading on a collagen matrix (Figure 1A). Adhesion localization of β-adaptin was observed in nascent, small adhesions that form at early stages of spreading (Figure 1A 45minutes) and it also localized to more mature, large adhesions in fully spread cells (Figure 1A 120minutes).

**Figure 2:**
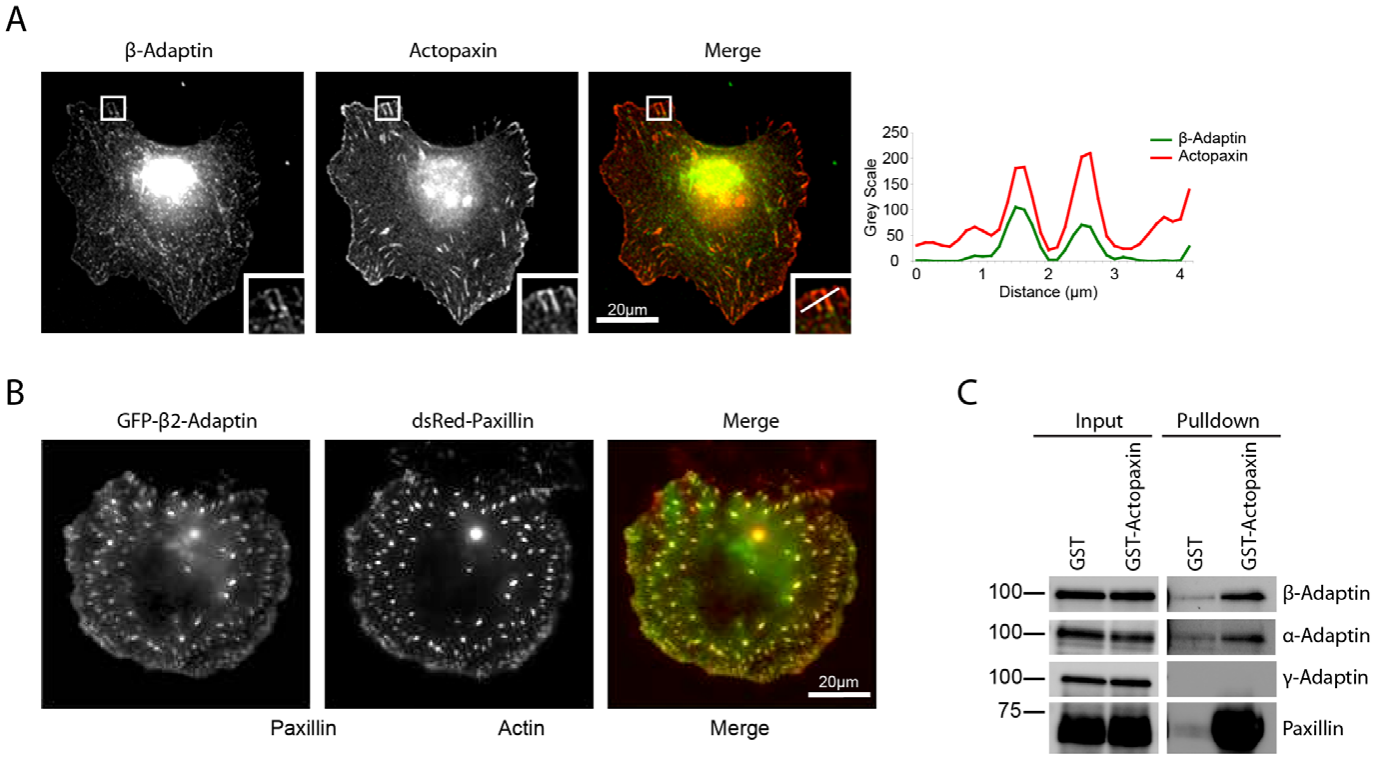
Actopaxin interacts selectively with the AP-2 complex. **A.** Endogenous β-adaptin and actopaxin colocalize at focal adhesions. Line profiles represent the localization of β-adaptin and actopaxin through the center of two adhesions. **B.** GFP-β2-adaptin localizes to focal adhesions. **C.** GST-Actopaxin precipitates a complex containing β- and α-adaptin, both subunits of the AP-2 complex but not γ-adaptin, a specific subunit of the AP-1 complex.

To identify which protein(s) facilitate the recruitment of β-adaptin to focal adhesions, a biochemical screen was conducted using GST-tagged focal adhesion scaffold proteins to pull down β-adaptin. This approach revealed that β-adaptin interacts with the focal adhesion protein actopaxin (Figure 1B) and the association of the two endogenous proteins at both 45 minutes and 120 minutes was confirmed by co-immunoprecipitation (Figure 1D). This interaction is direct as determined by *in vitro* binding experiments (Figure 1E). Actopaxin [22], also called α-parvin [25], is a focal adhesion-associated adaptor protein that binds to paxillin [22], ILK [26] and actin [22] and mediates cell spreading and migration [22], [27], [28]. We further characterized the interaction between β-adaptin and actopaxin and identified the N-terminal amino acid 1–95 fragment as being the region of actopaxin that binds β-adaptin (Figure 1C). Endogenous β-adaptin and actopaxin colocalized at focal adhesions (Figure 2A), while transfected GFP-β2-adaptin also localized to dsRed-paxillin-labeled adhesions (Figure 2B). Together, these data suggest that actopaxin may interact specifically with β2-adaptin and thus the AP-2 endocytic complex at focal adhesions, as opposed to β1-adaptin, which is a component of the AP-1 complex.

The AP-2 adaptor complex is responsible for cargo selection from the plasma membrane, while AP-1 is involved in cargo selection from the trans-Golgi network and endosomes [29]. The large subunits of the AP-2 complex are comprised of β2-adaptin and α-adaptin, while the AP-1 complex contains β1-adaptin and γ-adaptin. Western blotting of a GST-actopaxin pulldown demonstrated co-precipitation of β- and α- adaptin, but not γ-adaptin (Figure 2C), indicating that actopaxin does indeed interact preferentially with the AP-2 complex and not the AP-1 complex.

**Figure 3:**
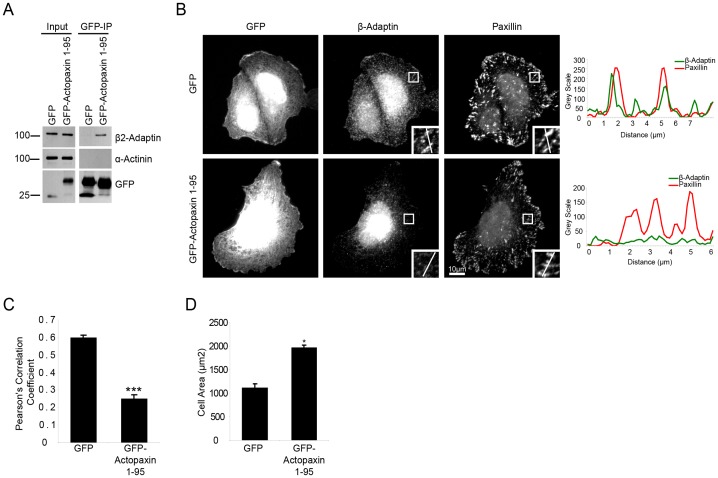
Beta 2-adaptin is recruited to adhesions through its interaction with actopaxin. **A.** Immunoprecipitation of GFP from U2OS cells expressing GFP empty vector or GFP-actopaxin 1–95 demonstrate an interaction between GFP-actopaxin 1–95 and β2-adaptin. **B.** U2OS cells expressing GFP or GFP-actopaxin 1–95 were spreading on collagen and stained for β2-adaptin and paxillin. In GFP expressing U2OS cells, β2-adaptin is targeted to adhesions. Beta2-adaptin and paxillin co-localize through adhesions as demonstrated by the line profile. When GFP-actopaxin 1–95 is expressed there is a loss of β2-adaptin from adhesions that remain positive for paxillin. Scale bar = 20 µm. **C.** Quantitation of β2-adaptin localization to paxillin positive adhesions using a Pearson’s correlation coefficient. Expression of the actopaxin 1–95 fragment significantly decreases β2-adaptin localization to adhesions. **D.** Quantitation of cell area of GFP empty vector and GFP-actopaxin 1–95 expressing cells. * = P<0.05.

To determine if β2-adaptin localizes to focal adhesions via its interaction with actopaxin, we generated a GFP-actopaxin 1–95 fragment that is able to bind β2-adaptin (Figure 3A), but cannot be localized to adhesions, as it lacks the paxillin and ILK binding sites of actopaxin [22], [26]. When U2OS cells were transfected with GFP alone, β2-adaptin localized to paxillin-positive adhesions (Figure 3B and C). In contrast, expression of GFP-actopaxin 1–95 resulted in a reduction in the localization of β2-adaptin to paxillin positive adhesions, as determined by immunostaining and quantification using the Pearson’s correlation coefficient (Figure 3B and C), while β2-adaptin localization to clathrin-coated pits was unaffected (Figure S1). Additionally, expression of GFP-actopaxin 1–95 resulted in an increase in cell area (Figure 3D). These data indicate that the localization of β2-adaptin to focal adhesions is likely mediated by its interaction with the amino terminus of actopaxin and that β2-adaptin localization to adhesions regulates cell spreading.

### β2-Adaptin Regulates Cell Spreading and Motility

**Figure 4:**
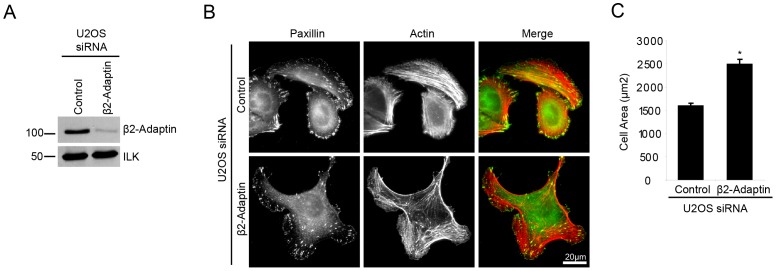
Beta 2-adaptin depletion results in enhanced cell spreading and lamellipodia formation. **A.** Western blot shows efficient siRNA-mediated knockdown of β2-adaptin in U2OS cells. **B.** Control and β2-adaptin siRNA U2OS cells plated on collagen for 120 min. Enhanced spreading and multiple lamellipodia formation is observed in β2-adaptin depleted cells. Scale bar = 20 µm. **C.** Quantitation of cell area of control and β2-adaptin siRNA U2OS cells. * = P<0.05.

To examine the role for β2-adaptin in adhesion-mediated responses, siRNA knockdown of β2-adaptin in U2OS cells was utilized (Figure 4A). Consistent with the over expression of GFP-actopaxin 1–95, which displaced β2-adaptin from adhesions, a significant increase in cell area was observed following β2-adaptin depletion compared to control cells when cells were spread on collagen for 120 minutes (Figure 4B and C). In addition to the larger cell area, β2-adaptin knockdown cells frequently displayed multiple lamellipodia (Figure 4B, Movies S1 and S2).

**Figure 5:**
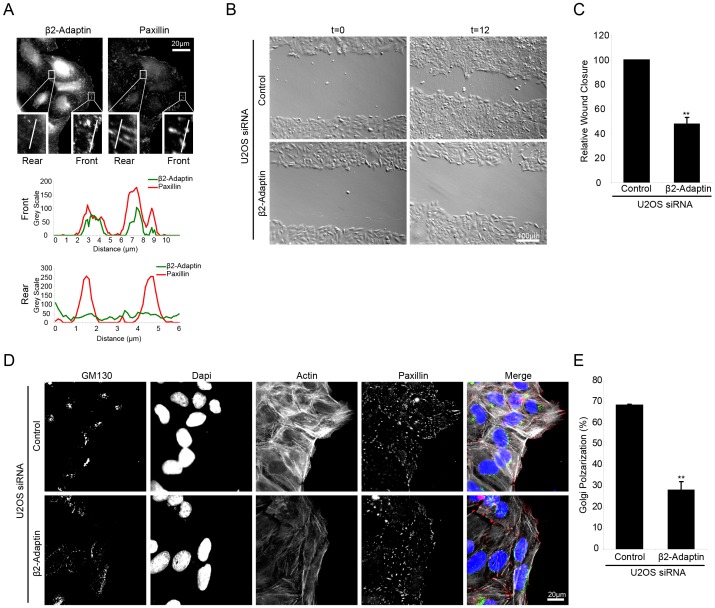
Beta 2-adaptin is required for directed cell migration. **A.** A confluent layer of parental U2OS cells was wounded and β2-adaptin localizes to paxillin adhesions at the leading edge of the cell but not at the rear as illustrated by line profiles. Scale bar = 20 µm. **B.** Confluent layers of control and β2-adaptin siRNA U2OS cells were wounded and allowed to migrate for 12 hrs. Beta2-adaptin knockdown results in decreased wound closure. Scale bar = 100 µm. **C.** Quantitation of wound closure. **D.** Control and β2-adaptin siRNA U2OS cells were wounded and allowed to migrate for 4 hrs prior to fixation. GM130 staining reveals non-polarized, fragmented Golgi in β2-adaptin depleted cells. Scale bar = 20 µm. **E.** Quantitation of polarized Golgi at the wound edge demonstrates a significant decrease following β2-adaptin knockdown. ** = P<0.005.

During directional cell migration, cells maintain a polarized morphology and typically extend a single lamellipodia at the leading edge. Given that siRNA knockdown of β2-adaptin resulted in the formation of multiple lamellipodia, this suggested that depletion of β2-adaptin may suppress the ability of cells to migrate in a polarized and directional manner. Immunolocalization of β2-adaptin in U2OS cells migrating directionally into a scrape wound revealed that β2-adaptin is enriched in paxillin-containing adhesions at the wound edge/leading lamellipodia (Figure 5A) and is largely absent from adhesions at the rear of the cell (Figure 5A). When β2-adaptin was depleted by RNAi, there was a reduction in wound closure compared to control siRNA treated cells (Figure 5B and C). To determine if the impaired wound closure was due, in part, to a defect in cell polarization, Golgi apparatus orientation at the wound edge was evaluated in response to RNAi depletion of β2-adaptin. At 4 hrs post wounding, control cells exhibited polarization of highly organized Golgi towards the wound edge, as visualized by staining for the cis-Golgi marker GM130 (Figure 5D). In contrast, β2-adaptin knockdown cells showed a significant decrease in polarization of the Golgi apparatus equivalent to that of random polarization (33%) (Figure 5E). These data indicate that β2-adaptin is required for directed cell migration through enabling proper front-rear cell polarization and Golgi apparatus orientation.

### Depletion of β2-adaptin Results in Matrix Degradation

**Figure 6:**
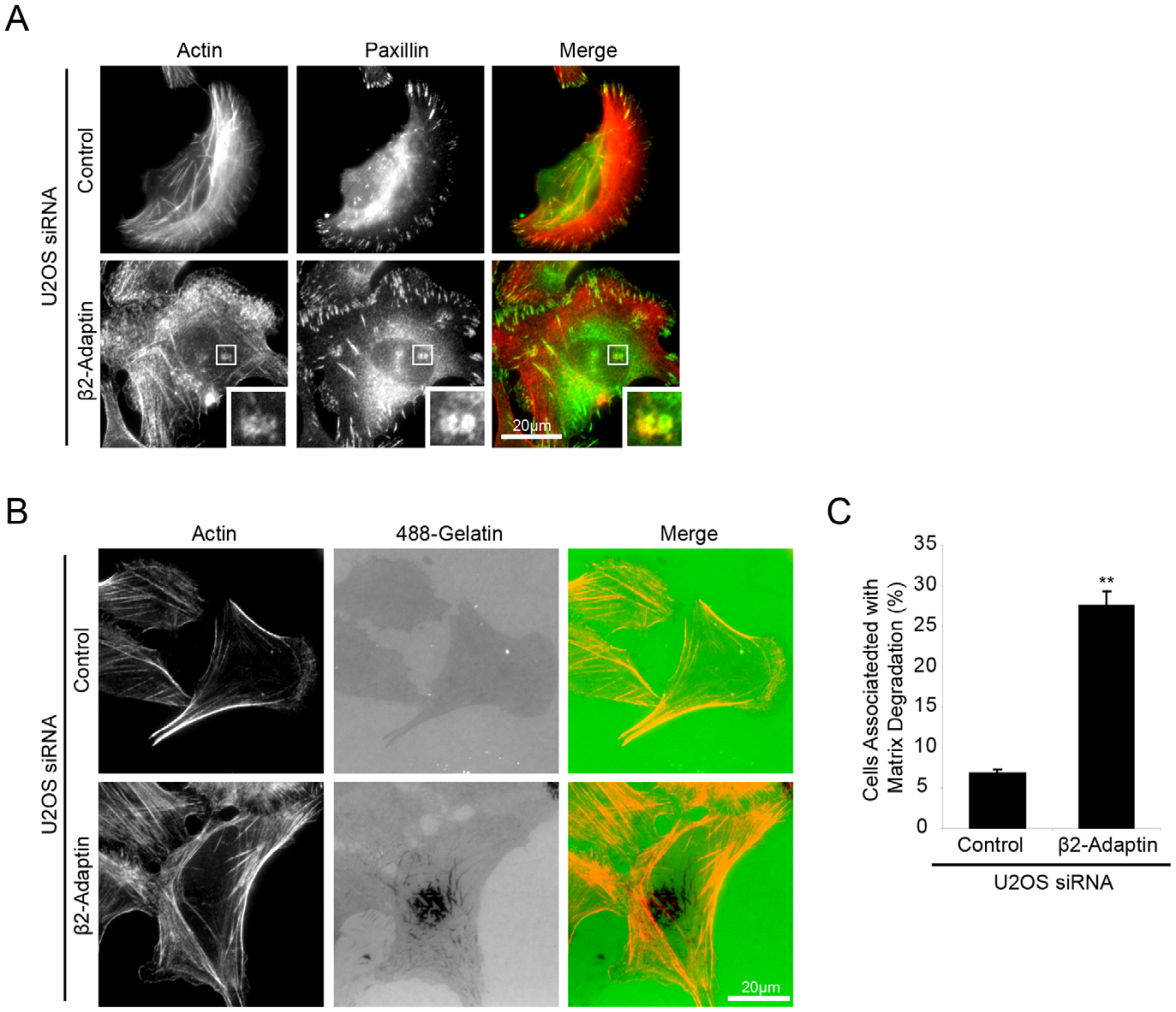
Beta 2-adaptin depletion induces matrix degradation in U2OS cells. **A.** U2OS cells depleted of β2-adaptin and spread on collagen form structures that appear similar to invadopodia, with paxillin and actin localized to ring and core-like structures respectively. Scale bar = 20 µm. **B.** When plated on fluorescent 488-gelatin, β2-adaptin depleted U2OS demonstrate increased ability to degrade matrix as compared to control cells. Scale bar = 20 µm. **C.** Quantitation of the number of cells degrading matrix shows a significant increase in β2-adaptin depleted cells. *** = P<0.0005.

While performing the initial characterization of cell spreading in control and β2-adaptin siRNA treated cells, macromolecular structures consisting of actin puncta surrounded by a ring of paxillin were observed, that were reminiscent of invadopodia (Figure 6A). Invadopodia are specialized adhesion structures that have the ability to focally degrade ECM, via localized matrix metalloproteinase (MMP) activity [20], [21] and are thought to coordinate ECM degradation and cell motility to facilitate the cell migration/invasion through the tissue stroma *in vivo* [21], [30], [31]. In order to assess if the invadopodia-like structures that formed following β2-adaptin depletion were functional and able to degrade extracellular matrix, cells were plated on fluorescent 488-gelatin for 16 hours, with a loss of fluorescence being indicative of MMP-induced gelatin degradation. Control siRNA treated U2OS cells do not readily degrade gelatin (Figure 6B and C). However, β2-adaptin-depleted cells displayed a significant increase in the ability to degrade gelatin matrix (Figure 6B and C), demonstrating that loss of β2-adaptin results in the formation of functional invadopodia in U2OS cells.

### β2-Adaptin Knockdown Induces the Formation of Invadopodia in Normal MCF10A Epithelial Cells

**Figure 7:**
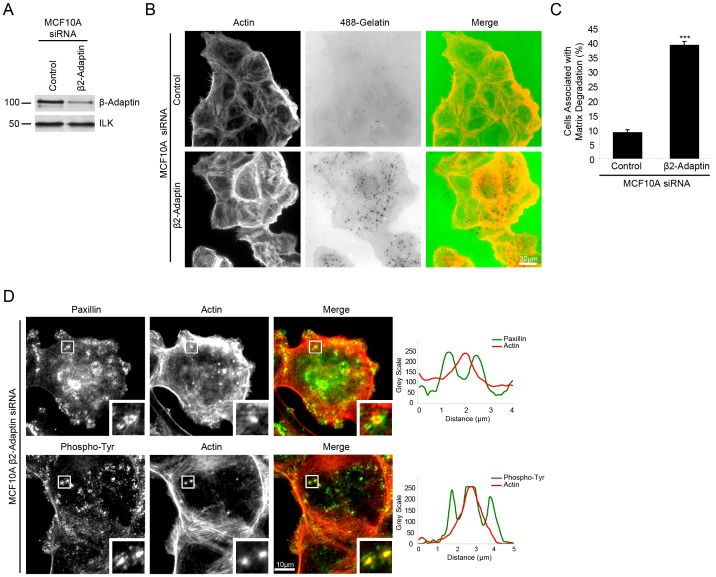
Beta 2-adaptin depletion in MCF10A cells induces invadopodia formation and matrix degradation. **A.** Western blot demonstrating efficient knockdown of β2-adaptin in MCF10A normal human breast epithelial cells. **B.** When plated on fluorescent 488-gelatin, β2-adaptin depleted MCF10A cells exhibit enhanced matrix degradation as compared to control RNAi treated cells. Scale bar = 20 µm. **C.** Quantitation of the percent of cells associated with matrix degradation shows a highly significant increase following β2-adaptin knockdown. *** = P<0.0005. **D.** β2-adaptin depleted MCF10A cells plated on collagen demonstrate the formation of invadopodia as seen by a paxillin ring staining surrounding actin cores, with phosphorylated tyrosine throughout the structures, as illustrated by line profiles. Scale bar = 10 µm.

The acquisition of an invasive phenotype is a key step in cancer progression. Furthermore, the ability of cancer cells to invade surrounding tissue and to metastasize correlates with the formation of invadopodia *in vitro* [32], [33], [34], The majority of human tumors are of epithelial origin. Therefore, we sought to determine if depletion of β2-adaptin by RNAi resulted in the formation of matrix degrading invadopodia in the non-invasive, normal breast epithelial cell line MCF10A. Indeed, β2-adaptin RNAi-depleted MCF10A cells (Figure 7A) exhibited a highly significant increase in matrix degradation as compared to control RNAi cells (Figure 7B and C). Staining of β2-adaptin-depleted MCF10A cells demonstrated the formation of paxillin-rich ring structures surrounding an F-actin-rich core that localized with sites of degradation. Furthermore, elevated phospho-tyrosine staining was localized throughout the structures, as illustrated by line profiles (Figure 7D), indicating distributions typical of invadopodia and demonstrating that in normal epithelial cells the loss of β2-adaptin results in the acquisition of these invasive structures.

**Figure 8:**
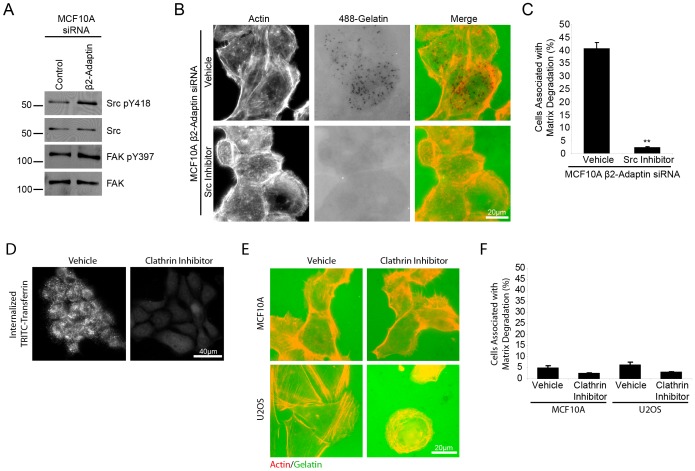
Beta 2-adaptin knockdown results in increased Src phosphorylation, which is required for matrix degradation. **A.** Western blot analysis of control and β2-adaptin siRNA treated MCF10A cells demonstrates β2-adaptin depleted cells have increased Src Y418 and FAK Y397 phosphorylation. **B.** Beta2-adaptin depleted MCF10A cells plated on fluorescent 488-gelatin in the presence of vehicle or Src inhibitor (PP2) show Src inhibition blocks matrix degradation. Scale bar = 20 µm. **C.** Quantitation of matrix degradation shows a highly significant decrease in matrix degradation when β2-adaptin knockdown MCF10A cells are treated with Src inhibitor. *** = P<0.0005. **D.** Treatment of MCF10A cells with MDC, an inhibitor of clathrin, results in an inhibition of TRITC-transferrin internalization. **E.** Treatment of MCF10A and U2OS cells with MDC fails to induce matrix degradation. **F.** Quantitation of matrix degradation of MCF10A and U2OS cells treated with vehicle or MDC shows no significant difference in the ability of cells to degrade matrix.

The requirement for Src-tyrosine kinase activity in the formation of invadopodia has been well documented [21], [35], phosphorylating a number of proteins necessary for the formation and function of invadopodia including cortactin [36], [37], ASAP1 [38] and p130 Cas [39]. Interestingly, Western blot analysis of β2-adaptin-depleted cells demonstrated an increase in Src activity when compared to control cells, as measured by blotting with anti-phospho Src Y418 (Figure 8A). An increase in FAK Y397 phosphorylation was also observed (Figure 8A). To determine if the increase in Src activity was required for β2-adaptin regulated matrix degradation, β2-adaptin knockdown cells were plated on fluorescent 488-gelatin in the presence of vehicle or the Src inhibitor, PP2 (Figure 8B). PP2 treatment abolished the ability of β2-adaptin knockdown cells to degrade matrix (Figure 8B and C). These data indicate that β2-adaptin may be involved in negatively regulating Src activity in non-transformed cells, as depletion of β2-adaptin resulted in increased Src activity. This in turn promotes invadopodia formation and matrix degradation in normal MCF10A, demonstrating a key role for β2-adaptin regulation of Src activity in maintaining a non-invasive phenotype in epithelial cells.

Global clathrin-mediated endocytosis can be inhibited with monodansylcadaverine (MDC) as shown using a TRITC-transferrin uptake assay (Figure 8D). Interestingly, inhibition of global clathrin-mediated endocytosis failed to induce matrix degradation in either the MCF10A or U2OS cells (Figure 8E and F), suggesting that the increased matrix degradation we observed following β2-adaptin knockdown is specific to perturbation of AP-2-dependent processes. In addition, these data suggest that the increase in Src activity and matrix degradation is not an indirect consequence of sustained growth factor receptor signaling.

## Discussion

In this study, the endocytic adapter protein β2-adaptin was shown to bind directly to the focal adhesion protein actopaxin. Furthermore, GST-actopaxin pull downs also contained α-, but not γ-adaptin, confirming a selective interaction with the AP-2 complex. Beta2-adaptin also localized to focal adhesions during cell spreading and migration in an actopaxin-dependent fashion. In contrast, localization of actopaxin to coated pits was not observed, suggesting that actopaxin may be involved in mediating AP-2 specific endocytosis at sites of adhesion via its interaction with β2-adaptin.

The interaction of β2-adaptin with actopaxin occurred within the N-terminal 1–95 region, that lacks the paxillin or ILK-binding site [22], [26] and when over expressed, this region of actopaxin does not localize to focal adhesions. This suggests that β2-adaptin localization to adhesions occurs indirectly via actopaxin binding to paxillin or ILK. Actopaxin is also an actin binding protein [22], but the functional significance of this interaction has not been evaluated. As actin plays a key role in the process of endocytosis [40], with a number of actin binding proteins such as Arp2/3, N-WASP, cortactin and mammalian Abp1p (mAbp1) being recruited to coated pits in mammalian cells [40], it is possible that actopaxin is playing a specific role in regulating focal adhesion associated endocytosis via the ability to link the clathrin endocytic machinery with the actin cytoskeleton.

Alternatively, actopaxin may be binding β2-adaptin, and therefore the AP-2 complex, in order to ensure that selective endocytosis of integrins and associated proteins occurs specifically at focal adhesions. A number of integrin subunits contain the internalization signal NPXY motif that is associated with clathrin-mediated endocytosis [41] and clathrin containing structures have been shown to facilitate microtubule-dependent focal adhesion disassembly and α5β1 integrin endocytosis following nocodazole washout [13], [16], [17]. Furthermore, αvβ5 integrin has been shown to localize to clathrin-coated pits [42]. The efficient disassembly and assembly of focal adhesions is a key event in directed cell migration [2] and the role for endocytosis in this process is just starting to be outlined. Therefore, the role of actopaxin and β2-adaptin in the regulation of focal adhesion dynamics warrants extensive further investigation.

Knockdown of β2-adaptin resulted in enhanced spreading and the formation of multiple lamellipodia in U2OS cells spread on collagen. Cells failed to polarize their Golgi when migrating into a scrape wound, demonstrating that β2-adaptin plays a key role in the establishment of cell polarity and consequently a single lamellipodium during migration. In migrating U2OS cells, β2-adaptin colocalized with paxillin in adhesions at the leading edge, but not at the cell rear, consistent with previous observations made with the μ2 subunit of AP-2 [19]. This suggests that AP-2-dependent endocytosis of adhesion proteins may be occurring preferentially at the leading edge, and indeed rapid endocytosis and recycling of receptors has been shown to occur at the front of migrating cells [4], [7], [8], [9].

Interestingly, post-endocytic trafficking of internalized integrin receptors has been shown to mediate persistent cell migration and lamellipodia formation via the re-delivery of integrins to the leading edge of migrating fibroblasts [43], [44]. In addition, endocytic trafficking of both Rac1 and Cdc42 is required for their recruitment from early endosomes to the plasma membrane and to the leading edge of migrating cells [45], [46] and this is dependent on the small GTPase Arf6. Downstream effectors of Rac1 coordinate actin dynamics and lamellipodia formation and Cdc42 regulates cell polarity [47]. Depletion of β2-adaptin may therefore be altering the localization and activity of Rac1 and Cdc42, resulting in aberrant lamellipodia formation and a loss of cell polarity. Interestingly, Cdc42 is recruited to the plasma membrane in association with its GEF β-PIX, which also interacts with actopaxin (Pignatelli J, Unpublished Data). Therefore, this suggests a possible role for β2-adaptin- and actopaxin-regulated endocytic trafficking in regulating polarized distribution of integrin receptors and small GTPases during directed cell migration.

In addition to the profound effect of β2-adaptin knockdown on polarized cell migration, depletion of β2-adaptin resulted in the formation of matrix-degrading invadopodia. Invadopodia are adhesion structures that occur in invasive tumor cells and other transformed cells. They consist of an actin core surrounded by a ring of adhesion-associated proteins, including integrins, Src, FAK, paxillin, vinculin, talin [21] and actopaxin [23]. They have the ability to degrade extracellular matrix via secretion and localized activation of proteases [20]. One of the key proteases in functioning invadopodia is MT1-MMP [48]. Clathrin-mediated endocytosis of MT1-MMP to lysosomal compartments controls proteolytic activity [49], [50], [51], [52] and this is also dependent on AP-2 [49], [50]. Prevention of MT1-MMP endocytosis results in an increase in surface levels and an increase in matrix degradation [53]. Therefore, β2-adaptin knockdown may be inhibiting AP-2-mediated endocytosis of MT1-MMP, resulting in increased surface levels of active MT1-MMP that may contribute to the formation of invadopodia and the increased matrix degradation observed in β2-adaptin depleted cells.

Knockdown of β2-adaptin resulted in activation of Src tyrosine kinase and inhibition of Src activity suppressed the ability of β2-adaptin knockdown cells to degrade matrix. Elevated Src expression and activity promotes the formation of invadopodia [21] and de novo invadopodia have been shown to form near to adhesions [54] in a Src-dependent manner. Inactive Src localizes to endosomes and active Src translocates to focal adhesions [55], [56], [57], [58], suggesting a key role for endocytic trafficking in regulating the spatial activation of Src. Knockdown of β2-adaptin may therefore cause changes in endocytic trafficking of Src, resulting in the elevation of Src tyrosine-kinase activity at focal adhesions and hence a transition to the formation of invadopodia. In previous studies, similar results were obtained following depletion of FAK in breast cancer cells, whereby spatial regulation of Src activity was altered and a switch of phospho-tyrosine proteins from focal adhesions to invadopodia was observed [59].

An alternative mechanism by which β2-adaptin depletion may result in the formation of invadopodia is via the regulation of Arf6-dependent trafficking. Arf6 is a small GTPase that binds the α-adaptin subunit of AP-2 [60]. Knockdown of α-adaptin or the μ2 subunit of AP-2 significantly reduces the amount of Arf6 observed at the plasma membrane [61]. Interestingly, Arf6 is localized to invadopodia and is crucial for invasion of breast cancer [62] and melanoma cells [63] and sustained activity of Arf6 enhances the invasive capacity of both of these cell types [62], [63]. As mentioned earlier, Arf6 redistribution of Rac1 and Cdc42 from early endosomes to the plasma membrane is also critical for the establishment of cell polarity and lamellipodia formation during directed cell migration [45], [46]. Therefore the potential link between actopaxin, β2-adaptin and Arf6 signaling warrants further analysis.

In summary, this paper demonstrates an interaction between β2-adaptin and the focal adhesion protein actopaxin and a role for β2-adaptin in regulating adhesion-mediated events including spreading and polarized cell migration. Depletion of β2-adaptin also results in the formation of invadopodia, suggesting that β2-adaptin controlled endocytic trafficking of specific cargo acts to regulate focal adhesion turnover and suppress the formation of invasive structures. Elucidating the molecular mechanisms by which actopaxin and β2-adaptin regulate trafficking and signaling of focal adhesion components will therefore provide critical insights into cellular events that contribute to a number of pathological situations.

## Supporting Information

Figure S1 **U2OS cells transfected with GFP-actopaxin 1–95 and dsRed clathrin heavy chain demonstrate that although β2-adaptin is lost from focal adhesions, it remained localized with non-adhesion associated clathrin-coated pits.**
(TIF)

Movie S1 **Control siRNA treated U2OS cells were seeded onto collagen in complete media and spread for 4 hours.** Time-lapse live cell images were taken every 2 minutes on a Nikon Eclipse Ti microscope using a 10X/0.30 PL FLUOR Nikon objective and Nikon NS-Elements software.
(AVI)

Movie S2 **Beta 2-adaptin siRNA treated U2OS cells were seeded onto collagen in complete media and spread for 4 hours.** Time-lapse live cell images were taken every 2 minutes on a Nikon Eclipse Ti microscope using a 10X/0.30 PL FLUOR Nikon objective and Nikon NS-Elements software.
(AVI)
