# Supplementary figures and images for: Beta2-Adaptin Binds Actopaxin and Regulates Cell Spreading, Migration and Matrix Degradation
Source: PLoS One. 2012 Oct 2;7(10):e46228. doi: 10.1371/journal.pone.0046228 (PMC3462795; doi:10.1371/journal.pone.0046228)

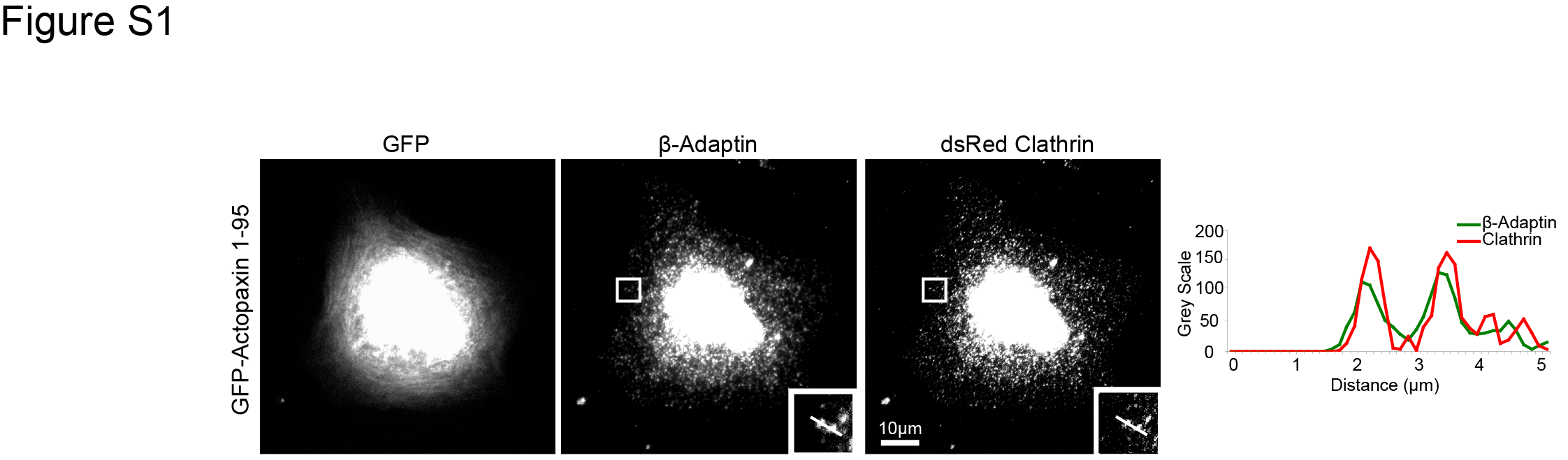

Supplement: Figure S1 — U2OS cells transfected with GFP-actopaxin 1–95 and dsRed clathrin heavy chain demonstrate that although β2-adaptin is lost from focal adhesions, it remained localized with non-adhesion associated clathrin-coated pits. (TIF) [file pone.0046228.s001.tif]
